# Microglia in Alzheimer's Disease: A Favorable Cellular Target to Ameliorate Alzheimer's Pathogenesis

**DOI:** 10.1155/2022/6052932

**Published:** 2022-06-02

**Authors:** Dewan Md. Sumsuzzman, Md. Sahab Uddin, Md. Tanvir Kabir, Sharifa Hasana, Asma Perveen, Ibtesam S. Alanazi, Ghadeer M. Albadrani, Mohamed M. Abdel-Daim, Ghulam Md Ashraf

**Affiliations:** ^1^Department of Pharmacy, Southeast University, Dhaka, Bangladesh; ^2^Pharmakon Neuroscience Research Network, Dhaka, Bangladesh; ^3^Department of Pharmacy, Brac University, Dhaka, Bangladesh; ^4^Glocal School of Life Sciences, Glocal University, Saharanpur, India; ^5^Department of Biology, Faculty of Sciences, University of Hafr Al Batin, Hafr Al Batin, Saudi Arabia; ^6^Department of Biology, College of Science, Princess Nourah bint Abdulrahman University, Riyadh 11474, Saudi Arabia; ^7^Department of Pharmaceutical Sciences, Pharmacy Program, Batterjee Medical College, P.O. Box 6231 Jeddah 21442, Saudi Arabia; ^8^Pharmacology Department, Faculty of Veterinary Medicine, Suez Canal University, Ismailia 41522, Egypt; ^9^Pre-Clinical Research Unit, King Fahd Medical Research Center, King Abdulaziz University, Jeddah, Saudi Arabia; ^10^Department of Medical Laboratory Technology, Faculty of Applied Medical Sciences, King Abdulaziz University, Jeddah, Saudi Arabia

## Abstract

Microglial cells serve as molecular sensors of the brain that play a role in physiological and pathological conditions. Under normal physiology, microglia are primarily responsible for regulating central nervous system homeostasis through the phagocytic clearance of redundant protein aggregates, apoptotic cells, damaged neurons, and synapses. Furthermore, microglial cells can promote and mitigate amyloid *β* phagocytosis and tau phosphorylation. Dysregulation of the microglial programming alters cellular morphology, molecular signaling, and secretory inflammatory molecules that contribute to various neurodegenerative disorders especially Alzheimer's disease (AD). Furthermore, microglia are considered primary sources of inflammatory molecules and can induce or regulate a broad spectrum of cellular responses. Interestingly, in AD, microglia play a double-edged role in disease progression; for instance, the detrimental microglial effects increase in AD while microglial beneficiary mechanisms are jeopardized. Depending on the disease stages, microglial cells are expressed differently, which may open new avenues for AD therapy. However, the disease-related role of microglial cells and their receptors in the AD brain remain unclear. Therefore, this review represents the role of microglial cells and their involvement in AD pathogenesis.

## 1. Introduction

Alzheimer's disease (AD) is a chronic neurodegenerative disorder that is well characterized by complex cellular and molecular alterations, such as loss of neurons and synapses, protuberant gliosis, dystrophic neuritis, formation of extracellular deposits of amyloid *β* (A*β*), and intracellular aggregated phosphorylated tau [1, 2]. Interestingly, reactive gliosis includes changes in function and morphology of astrocytes and microglia [3–5]. The neuroinflammatory process plays an important role in several neurological diseases, including autoimmune ailments [6]. In the case of AD development, the inflammatory response has been undoubtedly connected. Moreover, microglia have been found to have a pivotal role in the pathogenesis of sporadic AD [7–9]. M1 microglia produce inflammatory mediators, which cause inflammation and neurotoxicity, while M2 microglia produce anti-inflammatory mediators, resulting in anti-inflammatory and neuroprotective effects. Microglia-facilitated neuroinflammation is a dual-edged sword in neurodegenerative events, with both damaging and beneficial consequences [10].

Activated microglial cells surround the A*β* plaques during A*β* phagocytosis/compaction, which may play either a neuroprotective or neurodegenerative role that depends on microglial phenotype switching [11–14]. In fact, via a reduction in the levels of A*β* in amyloid precursor protein- (APP-) based models, chronic microglial activation might improve the AD pathology [15]. Nevertheless, it has been implicated that inflammatory response exerts harmful neurotoxic effects via the release of neurotoxins and proinflammatory chemokines/cytokines [16, 17]. Induction of inflammation is also likely associated with tau pathology [18]. Evidence suggests that microglia have been linked to tau pathology and spatial memory deficits [19].

Human genome-wide association studies (GWAS) further strengthened the relationship between microglia and AD pathology. GWAS data showed that the microglial immune response is associated with multiple polymorphisms [8, 20, 21]. On the other hand, within a diverse range of AD-related genes, the microglial triggering receptor expressed in the myeloid cell 2 (*TREM2*) gene appears to have a critical contribution in case of AD-related immune response [8]. TREM2 is a lipoprotein sensor and lipid that encourages reactive microgliosis via its DNAX activation protein of 12 kDa (DAP12, a transmembrane protein) [22, 23]. It has been exhibited that through its interaction with apolipoprotein E (APOE), TREM2 controls the transcriptional activation of microglial cells [24–26]. Nonetheless, the impact of TREM2-facilitated microglial activation in AD pathogenesis, or the activities of the microglial cell, is not yet well-explained [27].

In the case of AD individuals, the neuroinflammatory response is possibly not entirely beneficial or harmful. Indeed, an uncontrolled microglial reaction might be detrimental to the surrounding neuronal elements or neurons [28]. In AD mouse models, parabiosis experiments revealed that, with a negligible contribution of infiltrating macrophages, microglia are responsible for increasing the number of myeloid cells observed in brains with plaque pathology [29]. Furthermore, via the elimination of undesirable synapses and neurons (i.e., immature synaptic connectivity whereby less active synaptic connections are formed), microglia also contribute to the developmental sculpting of neural circuits [30, 31]. These microglial roles have been shown to be compromised in aging that contributes to AD progression [32–34]. Therefore, this review is aimed at discussing how microglia act as immune system cells and how this system is changed in AD pathogenesis.

## 2. Microglia in Brain Aging

Aging causes microglial morphology changes [35, 36]. It has been specified in mice that microglia surveying processes are not so dynamic and less critical because of age [32, 37]. This explains the impact of pathogenic response, response to accumulated protein, or delayed injury in aged brains rather than younger mouse brains. In a facial nerve axotomy study, microglial proliferation during aging remained significantly higher in response to neuronal injury, suggesting that regulation of microglial proliferation changes with aging [38]. Moreover, the migration rate of the microglial cell was affected by aging when microglia responded to injury [32, 39]. A study on the dynamic behavior and morphology of microglia with aging disclosed that microglial response significantly reduced with age [39], whereas the distal branches become thinner and contain major functions [40–42]. Most importantly, myelin fragmentation has a role in the formation of myelin inclusions [43]. In addition to this, aging can cause the reduction of the somatic volume of the microglial cell that reduces tissue distribution homogeneity [44, 45].

During aging, microglia show an increased inflammatory response and exhibit differential changes in expression level [35]. For example, the expression level of major histocompatibility complex (MHC) II and cluster of differentiation (CD) 68 was higher in the aged microglial cell [46, 47]. On the other hand, the CD200 shows a decreased expression [48]. In order to form the ramified microglia, the CX3CL/fractalkine cytokine also plays a similar role. CX3CL1 connects with C-X3-C motif chemokine receptor 1 (CX3CR1), which is expressed vastly in the microglial cell [49]. Generally, in the pathway of canonical signaling for transforming growth factor-beta (TGF*β*), Smad3 takes part in signaling, and the aged brain cell shows reduced anti-inflammatory functions [50]. Moreover, in proinflammatory gene transcription, interferon-gamma (IFN*γ*) activates microglia [51, 52], and the activation increases in the aged brain. Microglial maturation is influenced by altering gene function, which is predicted as a principal regulator of aging-associated changes in the microglial cell [53]. Apart from this, Iba-1 is highly expressed in microglia, which exerts its lessened ramified structure of microglial cells during aging [54], and it also accomplishes the proliferation of microglia.

According to the previous literature, microglial age-related phenotypes vary based on central nervous system (CNS) compartments [55]. The current studies have also reported that the aging effects on the microglial transcriptome are predominantly reliable on the basis of CNS locations [56]. Normally, microglia become highly activated during aging, and it acts towards CNS and peripheral nervous system (PNS) insults combined. Caldeira et al. [57] have reported by *in vitro* experiment that the isolated microglial cell tends to show a reduced reaction in autophagic capability, chemotaxis, phagocytosis, and overall reactivity.

## 3. Microglia in Neurodegeneration

Microglial activation exacerbates the production of cytokines, chemokines, and other factors that trigger AD progression [58]. Not only do proliferative microglia correlate with disease severity in AD patients but also AD animal models [59]. Their gradual gathering and changing in signaling prompt the cognitive decline; thereby, targeting microglia and their signaling pathways would be a potential therapeutic strategy.

By using a gene expression profile, a study identified a newer type of microglia that extended the existent microglia classification, and investigators in this inquiry yclept this molecular signature of disease-associated microglia (DAM, distinctive microglia subgroups) [24]. Interestingly, Krasemann et al. [24] showed that microglial neurodegenerative phenotype (MGnD) upregulated 28 inflammatory molecules and diminished the expression of 68 homeostatic microglial genes; in contrast, a large segment of these activities disappeared in the microglia-specific knockout of *APOE* in mice. These findings indicate that the *APOE* strongly persuades phenotypic switching in disease-related microglia and is upregulated through the vicinity of plaques. Moreover, MGnD microglia remarkably increased in miR-155 expression resulting in a notable upregulation of microRNA (miRNA) in microglia after extreme provocation with an insult, which leads to the release of proinflammatory molecules, including interleukin-6 (IL-6), interleukin-1*β* (IL-1*β*), nitric oxide synthase-2 (NOS-2), and tumor necrosis factor-alpha (TNF-*α*) [60]. In 5XFAD transgenic mice, elevation of *APOE*, *TREM-2*, and leukocystatin (*Cst7*) gene expression was associated with the transition from homeostatic microglia to DAM activation [61]. Previously, it has been demonstrated that DAM activation is tightly linked with the loss of microglial homeostatic genes such as purinergic receptor P2Y (P2RY12) and CX3CR1 [61].

In addition, Runt-related transcription factor 1 (RUNX-1), Sal-like 1 (SALL-1), T-cell-acute-lymphocytic leukemia protein-1 (TAL-1), and interferon regulatory factor 8 (IRF8) genes acquainted with microglia maturation and ramification are also influenced by AD pathology [60]. Usually, MGnD is a consequence of chronic manifestation of disease pathology and can easily differentiate between M1 and M2 microglia through the appearance of ApoE, TREM2, chitinase-3-like protein (Ym1), arginase 1 (Arg1) as well as the nonappearance of a homeostatic transcription factor, namely, early growth response protein 1 (Egr1), respectively [24]. In the AD brain, both the MGnD and DAM phenotypes are upregulated in the microglia and influenced mainly by *TREM2* expression [62, 63]. From these studies, the researchers propose that the *APOE*-*TREM2* signaling pathway is mainly accountable for the remodelling of the gene expression profile that prompts the MGnD phenotype in microglia [24, 61, 63]. While the relationship between the MGnD phenotype and aging is questionable, how microglia with advancing age are responsible for making this transition needs to be solved. Advance research is warranted for better understanding to elucidate the close relationship between the time-dependent *APOE*-*TREM2* signaling complex and the MGnD phenotype.

## 4. Activated Microglia and Alzheimer's Pathogenesis

**Figure 1:**
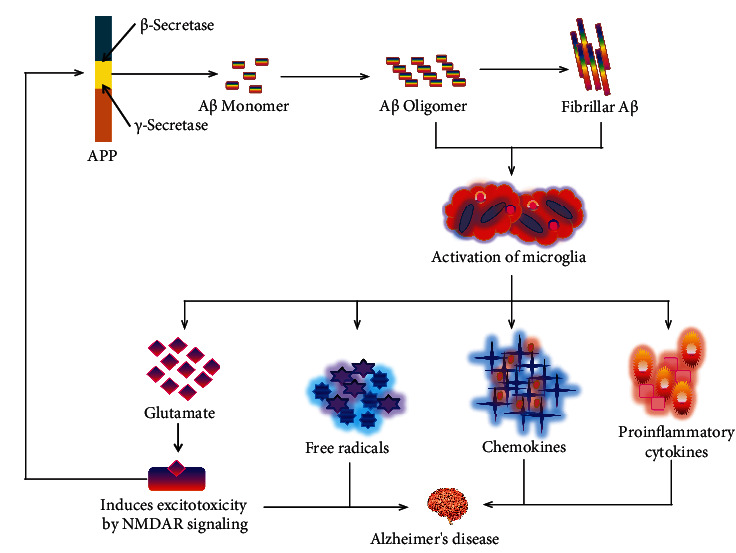
Role of A*β* in the activation of microglia to initiate Alzheimer's pathology. A*β*: amyloid beta; APP: amyloid precursor protein; IL-1: interleukin-1; IL-6: interleukin-6; TNF-*α*: tumor necrosis factor-*α*; MCP-1: monocyte chemotactic-1; MIP-1: macrophage inflammatory protein-1; HO: hydroxyl radical; H_2_O_2_: hydrogen peroxide; O_2_: oxygen radical.

Microglia have a dual role in AD pathogenesis; in AD, microglial detrimental effects are associated with proinflammatory mediators [64, 65]. Apparently, A*β* provoked the microglial activation, and deteriorated neuron-derived ingredients may exaggerate microglial neurotoxicity in AD [66]. A*β* subsists in several assembly forms, such as monomers, oligomers, and fibrils. However, from these three A*β* assemblies, only oligomeric A*β* (oA*β*) and fibrillar A*β* (fA*β*) have been implicated to microglial releases of proinflammatory mediators (Figure 1) such as cytokines (i.e., IL-1, IL-6, and TNF-*α*), chemokines (i.e., monocyte chemotactic-1 (MCP-1) and macrophage inflammatory protein-1 (MIP-1)), and reactive oxygen species (ROS) [67, 68].

The expression of nicotinamide-adenine-dinucleotide-phosphate-oxidase (NADPH oxidase) is stimulated to produce the ROS, which is correlated with the upregulation of AD [69]. fA*β* is liable to microglial NADPH [70, 71], and activation of NADPH oxidase eventually leads to neurotoxicity. In fact, microglia produce extracellular ROS that has been directly harmful to neurons. Moreover, intracellular ROS act like a signaling molecule in microglia, which promotes the secretion of different proinflammatory cytokines and neurotoxic molecules [72].

Furthermore, glutaminase expression was disorganized by microglial activation; as a result, release of a large proportion of glutamate influenced excitoneurotoxicity through the N-methyl-D-aspartate (NMDA) receptor signaling pathway [73–75]. Previously, it has been demonstrated that persistent triggering of extrasynaptic NMDA receptors contributes to accelerated A*β* production [76]. Accumulating evidence supports that expression of A*β* itself disrupts the synaptic function, such as suppressing hippocampal long-term potentiating, the assistance of prolonged depression, and disturbance of synaptic plasticity [77, 78]. Hence, it is crucially important to examine the microglial neurotoxicity along with A*β* neurotoxicity. In addition, both the tau protein and A*β* pathology have been directly linked to the neuroinflammatory responses through the accumulation of reactive microglia and astrocytes, which are close to the amyloid deposits, an additional histological characteristic of AD [8, 79]. For example, in P301S tau, transgenic mice exhibit prominent microglial activation that ultimately disrupts hippocampal synaptic function [80]. Thus, microgliosis-induced hippocampal synaptic pathology may be the earliest expression of neurodegenerative tauopathies. Activated microglia can also reactivate astrocytes by releasing cytokines, including IL-1*α*, TNF-*α*, and C1q [81]. Reactivation of these astrocytes notably upregulates complement cascade genes, including C3, and fails to contribute to synaptogenesis and phagocytose synapses and myelin debris. In the prefrontal cortex of AD patients, nearly 60% of the astrocytes are C3-expressing astrocytes and may possibly cause neuronal injury [81]. During AD, reactive astrocytes interact with neuronal and nonneuronal (i.e., microglia and oligodendrocytes) cells by secreting feedforward signals and contributing to the vicious cycle that expedites neurodegeneration [82]. Although reactive astrocytes have both beneficial and harmful functions during AD, atrophic astrocytes (reduction of the surface area and volume of astroglial morphological profiles) might lose their homeostatic functions.

Microglia-neuron communication is bidirectional. Microglia-derived exosomes serve as a carrier for tau and A*β* in the brain. On the other hand, neuron-derived exosomes have similar effects on microglia. A study has shown that microglia act as scavengers by uptaking neuronal exosomes containing toxic proteins, including pTau and A*β* [83].

## 5. Microglia Receptors in the Amyloid Cascade of Alzheimer's Disease

### 5.1. Complement Receptors

**Figure 2:**
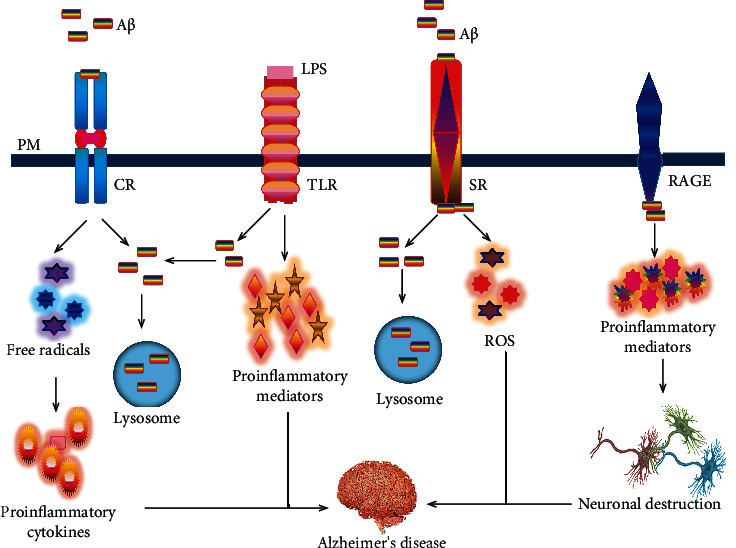
The linkage of microglia receptors in the pathogenesis of Alzheimer's disease. CR3 is responsible for the A*β*-induced microglial activation and involved in A*β*-mediated microglia free radical generation as well as uptake and clearance of A*β*. TLR2 is implicated in the generation of the inflammatory response. On the other hand, TLR4 (i.e., stimulated with LPS) is associated with the clearance of A*β*. Microglia cells showed an increase in A*β* uptake. The binding of A*β* to SRs internalizes A*β* and could activate inflammation responses and generate reactive species. Microglia RAGE-A*β* interaction triggers the genesis of proinflammatory molecules that causes neuronal destruction. PM: plasma membrane; A*β*: amyloid beta; CR: complement receptor; LPS: lipopolysaccharide; TLR: Toll-like receptor; SR: scavenger receptor; RAGE: receptor for advanced glycation end products; ROS: reactive oxygen species.

Complement components (CRs) and their receptors are categorized as cell surface molecules on microglia that are located within or around A*β* cerebral plaques in AD [84]. Previously, it has been demonstrated that microglia not only express complement protein components such as complement component-1 (C1q) and complement component-3 (C3) but also precisely express complement receptors, including complement receptor type-1 (CR1), complement receptor type 3 (CR3), complement receptor type-4 (CR4), and complement component 5a receptor 1 (C5aR1), which support phagocytic uptake [85]. The imbalance of these complementary systems is correlated with the development of AD pathogenesis (Table 1). For instance, A*β* plaque formation was observed to be markedly increased with the suppression of these complement systems in the AD transgenic mouse model [86]. However, different proteins of the complement system and its analogous mRNAs are unregulated, resulting in A*β*-instigated inflammation, the emergence of senile plaque, and A*β* phagocytosis in AD patients [87]. C3 is denoted as a protein and an integral part of the complement system, which influences the phagocytosis of pathogens by interacting with the CR3 receptor. CR3 is also familiar as a macrophage-1 antigen and indisputably observed in microglia that have been upregulated by the AD brains [88]. In addition, both *in vivo* and *in vitro* studies have demonstrated that CR3 was responsible for the uptake and clearance of A*β* [89–91]. Likewise, this receptor is partially associated with A*β*-induced microglial activation and involved in A*β*-mediated microglia ROS generation [92], as stated in Figure 2. Furthermore, a study in AD mice showed that microglia were associated with synaptic pruning in a CR3-dependent pathway [93]. More clearly, oligomeric A*β* locally activated complement (i.e., C1q and C3) at vulnerable synapses, resulting in microglial engulfment of these synapses via C3/CR3 signaling. Nowadays, CR3 antagonists are widely accepted as potential therapeutics to treat AD owing to their potential to significantly decrease the A*β*-induced proinflammatory molecules and ROS in microglia [92].

C5a is a protein fragment that can generate highly proinflammatory molecules via activating the complement system [41]. It is also called a CD88 and is located on the surface of the microglial cell. CD88 is involved in microglial recruitment and activation; an elevated level of CD88 has been observed in microglia and appeared close to the amyloid plaques in the AD mouse brains [119]. In addition, coinvigoration of human monocytes with A*β* and C5a encourages the promotion of IL-1*β* as well as IL-6 secretion [120]; mitigating the destructive role of CD88 would be a potential strategy for AD pathogenesis. Therefore, Fonseca et al. [97] conducted a study to assess the efficacy of this receptor antagonist, which markedly attenuated A*β* plaques, reduced glial triggering, and ameliorated context-dependent memory in double transgenic AD mouse models (Table 2).

Although accumulating evidence indicates the complement system manifested detrimental effects, a few data claimed that it has beneficial effects too in AD. For instance, C3-deficient APP mice showed an elevated level of A*β* in the brain area linked with notable neuronal damage [89]. More interestingly, higher expression of C3 mRNA levels is linked with a depletion in A*β* deposition in hAPP/TGF-*β*1 transgenic mice [121]. Overall, activation of these receptors might encourage the A*β* clearance, therefore eventually decreasing the A*β* accumulation in the AD. Still, many issues remain unsolved, so future studies are warranted to expurgate the molecular mechanism of the complement system in the brain and evaluate its suitability to the design and development of novel AD treatments.

### 5.2. Toll-Like Receptors

In 1997, Toll-like receptors (TLRs) were first identified as membrane proteins found in different types of cells, such as microglia and astrocytes [124, 125]. Although in mammals, there are 12 TLRs that have been described, only TLR2 and TLR4 can recognize A*β* [126]. However, its activation stimulates several signaling pathways; as well as, the secretion of several cytokines, nitric oxide (NO), and ROS [98]. Surprisingly, animal and human brain microglia expressed among the TLRs 1-9 and maxima of these receptors were responsible for microglial activation and neurotoxicity [125, 127]. For example, aged APP23 transgenic mice showed an upregulation of TLR-2, TLR-4, TLR-5, TLR-7, and TLR-9 mRNA levels in plaque-related brain tissue [128]. Studies have demonstrated that TLRs stimulate the intracellular cascade that leads to either release of proinflammatory mediators or the uptake and clearance of A*β* [99, 102]. Likewise, TLR2 involvement in the activation of microglial proinflammatory signaling to A*β* has been shown in Figure 2. Both AD patients and AD murine models found an increase in mRNA levels for TLR2 in the brains [129, 130]. Additionally, it has been reported that deficiency of TLR2 promotes a reduction in both spatial and nonspatial memory [123]. Interestingly, knockdown of TLR2 mice has disclosed a depletion of A*β*-induced manifestation of proinflammatory molecules (i.e., TNF-*α*, iNOS, IL-1*β*, and IL-6) and integrin markers (i.e., CD11a, CD11b, and CD68) in microglia [99]. Likewise, Liu et al. [101] have demonstrated that TLR2 deficiency suppressed A*β*-induced inflammatory signaling and improved A*β* internalization by phagocytosis in cultured microglia and macrophages. So suppression of TLR2 would be a powerful scheme that could markedly dwindle the inflammatory response and notably enhance the A*β* clearance, consequently slowing the AD pathogenesis.

TLR4 can recognize LPS by microglia; previous studies have identified its influences on stimulating the microglia-A*β* activation [131]. For instance, an activated murine microglia cell demonstrates that TLR4 contributes to A*β*-induced microglial neurotoxicity combined with a CD14 and myeloid differentiation protein-2 (MD2) [131]. In an *in vitro* experiment, microglia cells invigorated with LPS (i.e., a TLR4 ligand) showed an upregulation of A*β* uptake [102], as shown in Figure 2. In addition, both *in vivo* and *in vitro* studies on an LPS-deficient response have revealed that microglia increased the A*β* load and decreased A*β* uptake [102]. Moreover, in early stages, the TLR4-mutated AD animal model expressed a deficiency of spatial learning and increased levels of A*β*42 in the brain [122]. Altogether, roundup evidence on TLR2 and TLR4 indicates that depending upon diverse microglial phenotypes, these receptors have a complex role in AD. However, consolidated evidence strongly suggests that activation of TLR2 and TLR4 contribute to AD progression, and their inhibition may suppress AD pathogenesis [132]. Maybe these receptors show their beneficial effects in the early stages of AD, and their opposite role is exhibited in the late stages of AD due to diverse microglial phenotypes. Therefore, microglial TLR2 and TLR4 represent an acceptable target for therapeutic intervention within the disease progression, and targeting them could increase A*β* phagocytosis or reduce inflammatory responses [133–135].

### 5.3. Scavenger Receptors

Two kinds of scavenger receptors (SRs) have been identified in the CNS. Scavenger receptor type-A (SR-A) is manifested on microglia and astrocytes, whereas scavenger receptor type-B (SR-B) receptors are manifested on microglial and endothelial cells [108]. Microglial adherence via SR-A binding to fibrillar A*β* causes microglial immobilization, the genesis of ROS, and secretion of cytokines [70]. Both of these SRs could bind and internalize A*β* (Figure 2), inducing an inflammatory response that leads to AD pathogenesis [104]. Furthermore, both the SR-AI expression levels and A*β* clearance have been attenuated by prolonged preservation of microglia activation [106]. In addition, SR-AI deficiency with a presenilin1 (PS1)/APP transgenic mouse brain showed that increased levels of A*β* deposition correlated with an increase in mortality [11].

CD36 is a pattern recognition receptor (PRR) found on many different kinds of cells. This receptor comprehends not only exogenous molecules, for example, microbial elements [136] but also endogenous molecules, such as low-density lipoproteins (LDL), oxidized phospholipids (oxPCCD36) [137], programmed cell death-related cells, and A*β* [138]. CD36 is responsible for the development of several diseases, including AD [139]. Furthermore, CD36 interacts with A*β* by microglia to generate ROS [110] and activation in response to fA*β* [108, 110, 140]. For instance, reducing the expression of cytokine and chemokine such as monocyte chemoattractant protein-1 (MCP-1), IL-1*β*, macrophage inflammatory protein-1*α* (MIP-1*α*), macrophage inflammatory protein-1*β* (MIP1*β*), macrophage inflammatory protein-2 (MIP-2), and TNF-*α* has been seen in macrophages and microglia from CD36-deficient mice vivified with fA*β* [110]. However, in human brains, CD36 was observed at an overexpressed level with A*β* deposits, but without A*β* deposition, CD36 has not been detected in healthy brains [141]. Moreover, CD36 configures complexes with other PRRs to bind to fibrillar proteins.

CD163 is an unclassified SR that is expressed on mature tissue macrophages and is involved in hemoglobin-haptoglobin clearance from the blood [142]. CD163 engagement caused macrophages to produce proinflammatory mediators, indicating that CD163 is involved in macrophage activation [143]. Fabriek et al. [144] also reported CD163 functions as an innate immunological sensor and modulator of local inflammation in the host's defense against both gram-positive and gram-negative bacteria. Interestingly, CD163 was found to be expressed on microglia in the brains of patients with HIV-associated dementia [145]. However, whether CD163 is involved in AD pathogenesis is still elusive.

### 5.4. Receptor for Advanced Glycation End Products

The receptor for advanced glycation end products (RAGE) is a multiligand receptor and a compelling factor in aging that identifies the A*β* peptides [146]. Previously, it has been observed that A*β* provokes nuclear factor kappa light chain enhancer of activated B cell (NF-*κ*B) activation in several cells and stimulates the release of proinflammatory mediators by the dealings with RAGE [147, 148].

Different experimental data disclosed that microglial RAGE-dependent molecular signaling drives A*β*-induced inflammatory response and neuronal damage in the AD [112–114, 149]. In particular, the experimental result proposes that the p38 mitogen-activated protein kinase (MAPK) signaling pathways engage in the activation of microglia through the interaction between A*β* and RAGE receptor [113, 115]. Fang et al. [115] have documented microglial RAGE in the pathogenesis of AD and proposed that refraining of the RAGE signaling pathway may be a quintessential target for reducing the secretion of proinflammatory molecules like TNF-*α* and IL-1*β* after A*β* stimulation in the AD. Microglia RAGE-A*β* interaction stimulates to upregulate the proinflammatory response as a consequence; the neuronal destruction that directly influences a shortage in learning and memory is mentioned in Figure 2. Further exploration has been suggested to ascertain small molecules for the blocking of A*β*-RAGE interaction, which would be a possible therapeutic stratagem to deal with most devastating AD pathogenesis.

## 6. Microglia in the Spread of Tau Pathology in Alzheimer's Disease

The hyperphosphorylation and accumulation of microtubule-associated protein tau (MAPT) form the initial event before neurodegeneration [150]. In humans, neuroinflammation is positively linked with tau pathology and is involved in the production of tau hyperphosphorylation, accumulation, and neurodegeneration [151, 152]. In the P301S animal model of tauopathy, it has been shown that microglial activation is the earliest manifestation of tau pathology [80]. Notably, in this study, they administered FK506 (i.e., an immunosuppressant drug), which reduced the microglial activation and augmented the lifespan of tau (P301S) transgenic mice [80]. Later, Maphis et al. [19] demonstrated that activated microglia played a pivotal role in the proliferation of tau. Afterwards, Bolós et al. [153] reported that microglia phagocytose the tau. However, how microglia induced tau pathology is yet to be confirmed.

Interestingly, an in vivo humanized mouse model of tauopathy (hTau) showed that either chemical compound or genetically induced microglial triggering markedly manifested tau pathology and behavioral malformation [154]. Furthermore, in hTau mice, deficiency of microglia-specific CX3CR1 evolved in triggered microglial activation as a result of increased tau pathology and impaired working memory [154]. This effect is arbitrated through the IL-1/p38 MAPK signaling pathway. Another study showed that deleting CX3CR1 in hAPP mice promoted the expression of inflammatory mediators and enhanced plaque-independent neuronal abnormality as well as cognitive deficits [155]. The CX3CL1/CX3CR1 signaling pathway is an important neuron and microglial communication [156]. A study demonstrated that nonappearance of CX3CR1 weakens the microglial internalization of tau, which leads to AD progress [157]. Accumulating studies indicate that microglia-allocated neuroinflammation increases the tau pathology as a consequence of neurodegenerative disease. In hTau mice, Maphis et al. [19] evaluated that depending on the different disease stages, CX3CR1 deficiency is responsible for the onset and development of tau pathology. They suggest that these reactive microglia can influence the development of the tau pathology and be consistent with the propagation of pathological tau in the brain. In addition, a study reveals that lacking microglial TREM2 results in exacerbated tau pathology and a profound dysregulation of stress-related kinase pathways in a humanized mouse model of tauopathy [158]. On the other hand, TREM2 reduces neuronal tau hyperphosphorylation by reducing the microglial inflammatory response [159]. During the pathological investigation of human brains, it has been found that microglia morphologically degenerated and were associated with tau pathology [160]. These morphological changes are suggested to result from microglial senescence and chronologically precede the spread of tau pathology [161, 162].

## 7. Microglial Activation in Alzheimer's Stage

### 7.1. Activated Microglia in Early-Onset Alzheimer's

The amyloid cascade-neuroinflammation hypothesis characterized as an abnormal production of A*β* owing to the redundancy of A*β* synthesis or a dysfunctioning of A*β* clearance is the paramount causality of the AD, which consequently stimulates neuroinflammation-induced neuronal loss [163, 164]. Therefore, neuroinflammation is noticed as a critical factor in the development of AD pathogenesis [165]. In addition, activated microglia can be either proinflammatory or anti-inflammatory.

In AD at its early stages, it has been proposed that the initial microglial activation may have a beneficial function through the clearance of the amyloid and releasing potential nerve growth factors [166]. On the other hand, due to the failure of this process hence to promote the A*β* aggregation or other lethal products, therefore, activation of proinflammatory phenotypes leads to a rapid destruction of the neurons. However, the genetic data from GWAS propose microglial activation to be able to execute several critical functions in the early stages of AD and autonomous amyloid pathology [167, 168]. Although epidemiological analysis has demonstrated that people who take nonsteroidal anti-inflammatory drugs (NSAIDs) have an inferior frequency of AD, randomized control trials have not shown the effectiveness of these NSAIDs in subjects with later onset of AD [169]. Recently, one study hypothesized two particular stages of microglial activation in the AD trajectory, an early anti-inflammatory phase and an advanced proinflammatory phase [170]. In this case, targeting antimicroglial medications would be most favorable to protect against the battle of the proinflammatory phenotype in the advanced phase of this disease. In the early phase of AD, microglial activation is able to alleviate A*β* aggregation by augmenting its phagocytosis, clearance, and degradation properties [171, 172]. For instance, an investigation of amyloid plaques by electron microscopy demonstrated that microglia are efficiently engulfing A*β*, and A*β* appeared in the endosome-like cellular domain [173].

### 7.2. Activated Microglia in Late-Onset Alzheimer's

**Figure 3:**
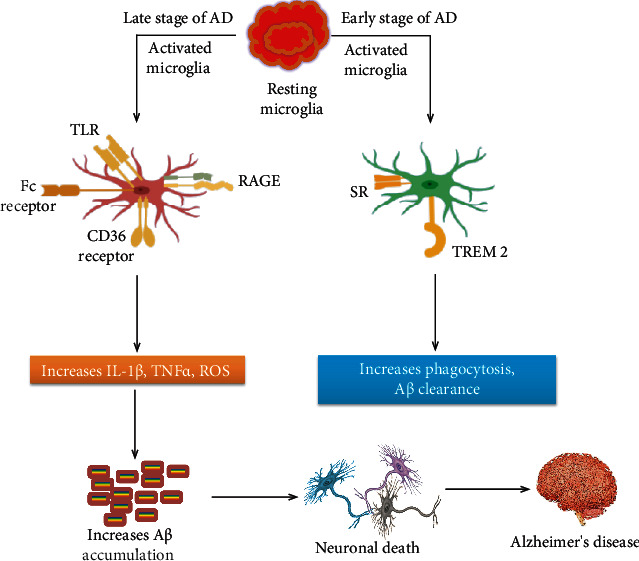
Possible mechanisms of action of activated microglia in different early and later stages of Alzheimer's disease. In the early stages of AD, activated microglia may increase A*β* clearance through TREM2 and scavenger receptors. On the other hand, in the late stage of the disease, continuous microglial activation induced by A*β* through various receptors triggers a vicious cycle of microglial activation, neuroinflammation, and A*β* buildup that leads to AD. AD: Alzheimer's disease; TLR: Toll-like receptor; RAGE: receptor for advanced glycation end products; IL-1*β*: interleukin-1*β*; TNF-*α*: tumor necrosis factor-*α*; ROS: reactive oxygen species; SR: scavenger receptor; TREM2: triggering receptor expressed on myeloid cell 2

In late-onset AD (LOAD), microglia have been deprived of their beneficiary function due to a tenacious production of proinflammatory mediators [174]. A study has shown that the amyloid plaque burden upsurges with aging in human patients, indicating the relatively ineffective phagocytic potential of microglia [175]. In human AD, A*β*42 immunization improves the function of microglia by intensifying their phagocytic activity [176]. Based on the microglial dysfunctioning notion, there is a loss in microglial neuroprotective activity in AD, rather than an increase in an inflammatory role [177]. Previously, it has been reported that the microglial phagocytic capabilities are shifted with aging and similarly decrease this feature in neurodegenerative diseases. Likewise, these senescent (i.e., biological aging) microglia are linked with the onset of sporadic AD [178]. Furthermore, recent studies on TREM2 also ascertain both early and late stages of microglial activation during the AD trajectory [179, 180]. TREM2 expressed in microglia is supposed to link with microglial activation. Even though a few studies indicate opposed effects for TREM2 levels in AD, lately, a study reported the level of soluble TREM2 (sTREM) directly linked with the early and delayed stage of AD [181, 182], where the peak beneficial role of TREM2 has observed in the early stage and later stage; its salutary effect gradually decreased (Figure 3).

## 8. Microglial Deterioration in Alzheimer's Patients

The activated microglial response has been extensively explored in AD brain regions by comparatively exalted A*β* subjects or in A*β*-rich transgenic models [183–185]. Fascinatingly, A*β* accumulation and neurofibrillary tangles (NFTs) do not appear in similar anatomical locus; in this sense, a direct pathogenic connection between amyloid plaques and neurodegenerative diseases is still elusive [186, 187]. In fact, cognitive disability is not compatible with an overabundance of amyloid plaque, even so with the presence of neurofibrillary pathology explicit as tau-positive morphology, including unmyelinated axons in the nervous system so-called neuropil threads, NFTs, and neuritic plaques [188, 189]. Additionally, an unambiguous determination of microglial activation in the human brain is extremely complicated since there is no effective biomarker for differentiating between activated and nonactivated cells. It is also surprising that microglial cells become progressively dysfunctional with aging in the human brain that displays morphologically senescence rather than activation, like fragmented cytoplasmic processes [190]. The identification of senescence microglia has imparted new aspects on the possible implication of microglia in aging-associated neurodegeneration; for example, aging causes loss of notable microglial cell function involved in the reduction of microglial neuroprotection [190, 191]. A study evaluating the microglial reaction in postmortem hippocampal human tissue demonstrated that microglia underwent a noticeable degenerative process in the dentate gyrus (DG) as well as CA3 of Braak V–VI samples, likely to be the case linked with the accumulation of soluble pTau [160].

Not only are microglial cells able to protect the synaptic integrity [192] but also they contribute to the learning ancillary synaptic formation [193]. Moreover, microglia induce A*β*-phagocytosis [194, 195] and senile plaque compaction and limit the A*β* toxicity [196, 197]. Furthermore, microglia contribute to removing depreciated neurons as well as neuronal stuff, for example, paranormal synaptic terminals or axonal demyelination. In this context, deficits in colony-stimulating factor 1 receptor (CSF1R) or TREM2 are correlated with a rare group of neurodegenerative disorder, for example, adult-onset leukoencephalopathy with axonal spheroids (i.e., characterized by excessive demyelinating lesions in the cerebral white matter) or Nasu-Hakola disease (i.e., characterized by multiple bone cysts linked to neurodegeneration), respectively [198–200]. These studies, together with Sanchez-Mejias [160] data, strongly indicated microglial pathology resulting in a deficient immunoprotection in DG and CA3 that leads to progressive AD pathology and cognitive damage. Furthermore, TREM2-knockout models show dystrophic microglial cells [201], shortages in microglial survival, and worsening in AD pathology [22].

Accumulating evidence demonstrates that AD is associated not only with microglial activation but also with microglial senescence, which might be considered the degeneration of these cells continuously [190]. These findings suggest that NSAIDs have become incapable of preventing or decreasing neurodegenerative disease like AD. Surprisingly, they reconstructed the conception regarding AD pathogenesis far away from inflammation-related impairment and proximate to an uninvestigated area of neuroscience, for instance, activities or events that can destroy microglial cells. Incredibly, it has become crystal clear that senescence microglia are responsible for age-related telomere length (TL) shortening [202, 203]. In addition, shortened TL in peripheral blood leukocytes is further recognized as early jeopardy of dementia [204]. Since microglia are indispensable for providing neuroprotection [191], aging-associated loss of a microglial protective role in neurodegenerative disease is likely to have detrimental repercussions for neurons.

## 9. Conclusion

Nowadays, studies started focusing on microglia to better understand the functional role of microglia in changing the progression of AD. Microglial cells are dynamic and reactive and change their surrounding environment rapidly, resulting in either proinflammatory or anti-inflammatory states. It has become apparent that microglia not only produce neurotoxic products but also need for phagocytic clearance of neurotoxic proteins associated with AD. The randomized control trials that employed nonspecific anti-inflammatory agents have not appeared to be significant in mitigating disease, possibly because of the inhibition of indispensable phagocytic functions that accumulate toxic proteins. Furthermore, depending on the clinical environment, microglia phenotypes may have a negative or positive effect. In fact, the ultimate beneficial role of TREM2 has been observed in the early stage and later stage, and its beneficial effect gradually decreased. AD pathogenesis is dependent on microglial cells and their receptors. Therefore, targeting microglial receptors to maintain microglial homeostasis would be a potential therapeutic strategy in AD.

## Figures and Tables

**Table 1:** Outline of microglia receptors and their function in Alzheimer's disease.

Microglia receptors	Functions in Alzheimer's disease	References
Complement receptors (CRs)	(i) Phagocytic uptake (ii) Microglia activation (iii) Proinflammatory molecule generation (iv) A*β* clearance	[89–92, 94–97]
Toll-like receptors (TLRs)	(i) Proinflammatory mediator generation (ii) A*β* clearance (iii) Microglia activation (iv) Synaptic plasticity (v) tau phosphorylation	[15, 98–103]
Scavenger receptor type-A (SR-A)	(i) A*β* internalization and clearance (ii) Inflammatory response (iii) Maintain microglia immune response	[104–107]
Cluster of differentiation 36 (CD36)	(i) Microglia recruitment (ii) Inflammatory response (iii) Activation of A*β* phagocytosis (iv) Modulates microglial A*β*42 phagocytosis	[108–111]
Receptor for advanced glycation end products (RAGE)	(i) Microglia activation (ii) Stimulate IL-1*β* (iii) TNF-*α* production (iv) Intensify oxidative stress	[112–116]
Triggering receptor expressed in the myeloid cell 2 (TREM2)	(i) A*β* clearance (ii) Regulates microglial mammalian target of rapamycin (mTOR) activation and metabolism (iii) Balanced microglial autophagy	[117, 118]

**Table 2:** Microglia in various Alzheimer's disease preclinical models.

Species/studied material	Experimental model	Effects	References
Complement C3-deficient APP transgenic mouse (APP; C3^−/−^)	Mouse model of AD	(i) Increased A*β* levels (ii) Elevation of fibrillar amyloid plaque burden	[89]
Homozygous C3-deficient and Mac-1-deficient mice (C3^−/−^; Mac-1^−/−^)	Mouse model of AD	(i) Implicated in the phagocytosis and removal of fA*β* by microglia	[91]
hAPP transgenic mouse	A*β*-induced neurotoxicity	(i) Reduced A*β* accumulation	[121]
TLR2 knockdown mice	A*β*42-induced neuroinflammation	(i) Suppressed proinflammatory molecules and integrin markers in microglia	[100]
Mutation of TLR4 in C3H/HeJ mice	A*β*-mediated cognitive dysfunction and neurotoxicity	(i) Reduced microglial activation (ii) Increase A*β* deposits	[122]
CD36-deficient C57BL/6J mice	Nitro blue tetrazolium induced ROS generation	(i) Decreased microglial recruitment to sites of fA*β* (ii) Reduced the production of ROS, TNF-*α*, IL-1*β*, and several chemokines	[110]
RAGE overexpressed mAPP transgenic mice	Mouse model of AD	(i) Increased the production of IL-1*β* and TNF-*α* (ii) Enhanced the infiltration of microglia as well as astrocytes (iii) Decreased acetylcholine esterase activity and causes A*β* accretion	[115]
Mutation of TLR4 in Mo/Hu APPswe PS1dE9 mice	Amyloidogenesis	(i) Increases in diffuse and fA*β* deposits (ii) Decreased A*β* uptake by microglia	[102]
PS1-APP transgenic mice	Aging induced A*β* deposition	(i) Early microglial enrollment fosters A*β* clearance (ii) A*β*-mediated inflammation reduces microglial A*β* clearance	[106]
Aged APP23 transgenic mice	A*β* induced neuroinflammation	(i) TREM2 is increased in microglia associated with amyloid plaques (ii) Lack of extracellular amyloid clearance due to TREM2 signaling	[117]
AD-mutant hAPP transgenic mice	Complement receptor and microglia mediated synaptic loss early in AD	(i) Inhibition of CR3 decreases phagocytic microglia (ii) Adult brain microglia engulf synaptic material when exposed to soluble A*β* oligomers	[93]
Triple transgenic mice that are deficient in TLR2 (TLR2^−/−^)	A*β*42-induced memory impairments	(i) Accelerated spatial and contextual memory impairments (ii) Increased levels of A*β*	[123]
APP/PS1/SR-A^−/−^ mice	A*β* induced cognitive decline and neuroinflammation	(i) Increased neuroinflammation (ii) Elevated A*β* accumulation (iii) Increased cognitive impairment	[107]
